# Design and damping characterization of sandwich composite made of particle-filled hollow spheres and steel sheets

**DOI:** 10.1038/s41598-024-76714-1

**Published:** 2024-10-25

**Authors:** Xin Zhou, Lars Penter, Ulrike Jehring, Hartmut Göhler, Thomas Weißgärber, Steffen Ihlenfeldt

**Affiliations:** 1 Chair of Machine Tools Development and Adaptive Control, Institute of Mechatronic Engineering, Technische Universität Dresden, Dresden, Germany; 2 Fraunhofer Institute for Machine Tools and Forming Technology IWU, Chemnitz, Germany; 3 Fraunhofer Institute for Manufacturing Technology and Advanced Materials IFAM, Branch Lab Dresden, Dresden, Germany

**Keywords:** Particle-filled hollow spheres, Sandwich composite, Machine tool, Design of experiment, Material characterization, Damping, Structural materials, Materials science, Engineering, Mechanical engineering

## Abstract

Sandwich composites made of particle-filled hollow sphere structures (PHSS) and steel sheets offer excellent lightweight and damping properties, ideal for high-speed machine tool components under dynamic loads. Previous research has focused on single particle-filled hollow sphere (PHS) and small PHSS, leaving a gap understanding of PHSS/steel sandwich composites. In this study a test rig was developed, and Design of Experiments and Response Surface Analysis were used to investigate the effects of sheet and core thickness (design parameters), filling ratio, and particle size (particle parameters) on the damping performance of PHSS/steel sandwiches. The results indicate that the design parameters have a significant influence on damping performance. The interaction between design and particle parameters also substantially affects damping. Minimizing particle size, increasing filling ratio, thinning the face layer, and thickening the core layer significantly improve structural damping. To address manufacturing tolerances, a finite element (FE) model-based optimization was developed to accurately determine PHSS material parameters. These parameters were used in an FE model of the PHSS-steel composite, with identified contact parameters minimizing measurement and simulation differences. The homogenized material model and the linear model using global damping parameters accurately reproduce the dynamic properties of the PHSS-steel sandwich composite in low vibration modes.

## Introduction

Machining operations in current machine tools are often performed at high feed rates to maximize the productivity. The increased dynamics of moving components typically result in large acceleration changes, which can reduce the machining accuracy. Machine tool components should be designed with high stiffness, low mass, and high damping capacity to reduce critical vibration amplitudes during highly dynamic structural excitations^1^. It is difficult to successfully integrate lightweight design and sufficient damping with current construction materials such as steel, cast iron, and aluminum. The development of novel technologies is necessary to solve this issue.

Compared to active damping techniques, which require additional equipment, passive damping methods can be utilized in mechanical engineering, including inherent approaches such as tuned-mass-damper^2^ and designed-in material damping^3^, without additional energy input. Particle damping offers a great opportunity to improve structural damping without requiring large or complicated components^4^. Particle dampers are typically hollow bodies filled with metallic or ceramic particles. They effectively reduce vibration by being attached to a structure where the maximum vibration amplitude occurs^5,6^. The majority of particle dampers are made by filling a small amount (less than 1000) of centimeter- or millimeter-sized particles into one or more cavities (less than 100)^7^. According to Meyer et al., the gap between the particle and the enclosure affects the maximum loss factor, and a high filling ratio and small particle size are desirable to increase the damping of a single particle damper. Particle stiffness, contact parameters, and gravity have little effect on the loss factor but the material of the filled particles does^8^. Koch et al. developed a honeycomb structure filled with different particles and came to a similar conclusion by studying the damping behavior of an oil pan whose bottom plate was replaced by a particle-filled honeycomb structure^9^. Filling granular material shows greater potential in vibration damping than liquid^10^. According to Gnanasambandham et al. the damping capability of a particle damper can be improved by dividing the hollow body into several parts, even at low excitation forces^11^. Jadhav et al. compare single-cell and multi-cell particle dampers by attaching them to the end of a cantilever beam. It is shown that a damper performs better when it has multiple cells filled with particles. Cell size and particle mass have significant effects on damping performance. Damping increases with growing cell size until an appropriate mass ratio is reached, after which the damping performance remains nearly constant. Inefficient energy dissipation can occur in a particle damper with a small cell size and a high mass ratio because momentum transfer between particles is limited^12^. The single particle dampers only affect the target vibration mode and have a position-dependent damping capability^13^.

Particle-filled hollow sphere structures (PHSS) consist of millimeter-sized hollow spheres filled with loose micrometer-sized particles and bonded together by sintering, soldering, bonding, or casting. It combines structure-integrated, adaptive passive damping with a high lightweight factor by utilizing a sandwich design. As a result, the PHSS/steel sandwich composite allows greater flexibility in the structural design and optimization of highly dynamic moving assemblies. Solid cover sheets provide the required stiffness, while a PHSS core ensures the necessary damping characteristics through internal dissipation processes^14^. The particle dampers investigated so far mainly consist of single particle dampers or dampers with large cell or that are divided into a few cells filled with large particles. Compared to a standard particle damper, the PHSS contains many tiny cells and an enormous number of particles. Instead of being concentrated at a discrete point as in single particle dampers, the vibration energy absorbed by PHSS is uniformly distributed throughout the material due to its layered and cellular structure. The previous work^15^ investigated the properties of single particle-filled hollow sphere (PHS) and PHSS, it is observed that as the particle diameter increases, the damping of PHS decreases and the damping is not affected by the size and thickness of the hollow sphere. It is not clear how the particle and design parameters of PHSS/steel sandwich composite affect its damping capability. In a complicated multi-material design, damping can be influenced by a variety of factors, including material properties, joint stiffness, and the way they are clamped.

Several experimental methods have been used to evaluate the damping properties of sandwich structures using a modal exciter. Mead^16^ compared different techniques for a damped beam. He shows that for a highly damped sandwich beam, the half bandwidth method (HBM) and energy measurement by resonance are still accurate. The study of Li and Cocker^17^ investigated the effect of thickness of the sandwich core and the cover layer on the damping. The damping of the sandwich grows as the core thickness increases. The low-frequency and high-frequency damping of the sandwich reduces as the face sheet thickness decreases. Using cross-linked fibers as the core material, Piollet et al.^18^ presented a test setup to quantify the loss factor of a sandwich beam. In this investigation, the sandwich beam was fixed at one end and the damping characteristics were evaluated using HBM. The stiffness and damping characteristics of this sandwich beam show a dependence on the amplitude. Michon et al.^19^ provided a test configuration for measuring an aluminum honeycomb cantilever beam filled with particles. The filled beam demonstrated viscous damping characteristics, which depend on the filling ratio. These studies used constrained boundaries to investigate the properties of the material fixing them at one end. The clamping condition has a significant effect on the dynamic properties of a composite structure^20^. To replicate an unconstrained boundary, Kadioglu et al.^21^ developed a test setup to measure glass-reinforced aluminum sandwich structures by mounting the specimen directly on the modal exciter. Peters et al.^22^ proposed a similar test setup where the sandwich beam is mounted in the center of a shaker table. However, for certain vibration modes, the maximum displacement of the specimen occurs where the mounting point is located and the mounting has considerable stiffness. In this case, certain vibration modes become unexcitable and do not form an unconstrained boundary in the measurement. This can potentially lead to inaccurate results and may cause loss of relevant information about the dynamic properties of the sandwich structure.

Before PHSS/steel sandwich composites can be widely used in machine design, several shortcomings need to be addressed.

1. Component design requires accurate numerical models that reproduce the static and dynamic properties of PHSS within manufacturing tolerances. This requires an appropriate method to obtain the mechanical material parameters of PHSS. Due to the cellular structure and unavoidable manufacturing errors, traditional static methods for determining mechanical material parameters were influenced by the interface between the PHSS and the test rig, while the non-destructive ultrasonic method is not applicable to PHSS with filled particles.
2. Previous studies lack a comprehensive investigation on PHSS/steel sandwich for their design (sheet and core thickness) and particle parameters (particle size and filling ratio) on its damping characteristics.
3. Previous experimental studies and testing strategies on sandwich structures do not adequately consider the influence of the test configuration, equipment, and boundary conditions on the dynamic properties of the test specimens. As a result, the obtained dynamic material behaviors do not accurately represent the true characteristics of the sandwich structures.

The objective of this study is to develop an accurate approach to describe the characteristics of PHSS as well as PHSS/steel sandwich composites, and to understand how design and particle parameters influence the damping performance of the PHSS/steel sandwich composite using design of experiments and statistical analysis. The authors propose a homogenized material model in combination with an optimization algorithm that iteratively adjusts the model parameters to best fit the measured data to identify the material parameters of PHSS. The material model is applied to an FE model of the sandwich composite with contact parameters identified by experimental data. The FE composite model integrates a linear damping model to simulate the dynamic properties.

Section 2 of the paper provides a comprehensive description of the fabrication technique used for PHSS and PHSS/steel sandwich composites and the characterization of the parameters of PHS. Section 3 presents the mechanical material parameter characterization of PHSS used in this study. Section 4 gives an overview of the experimental configurations investigating the damping characteristics of PHSS/steel sandwich composites and the test specimen design for response surface analysis determining the effect of design and particle parameters on the damping. Section 5 shows the FE-model of the sandwich composite with identified material parameters of PHSS from Sect. 3 and global damping model to simulate the frequency response of PHSS/steel sandwich composite, and the comparison of the simulation with corrected measured FRF by eliminating the external effect caused from test rig.

## PHSS/steel sandwich composite

This section illustrates the fabrication process and technique for PHSS and PHSS/steel sandwich composite.

### Fabrication of PHS, PHSS and PHSS/steel sandwich composite

**Fig. 1:**
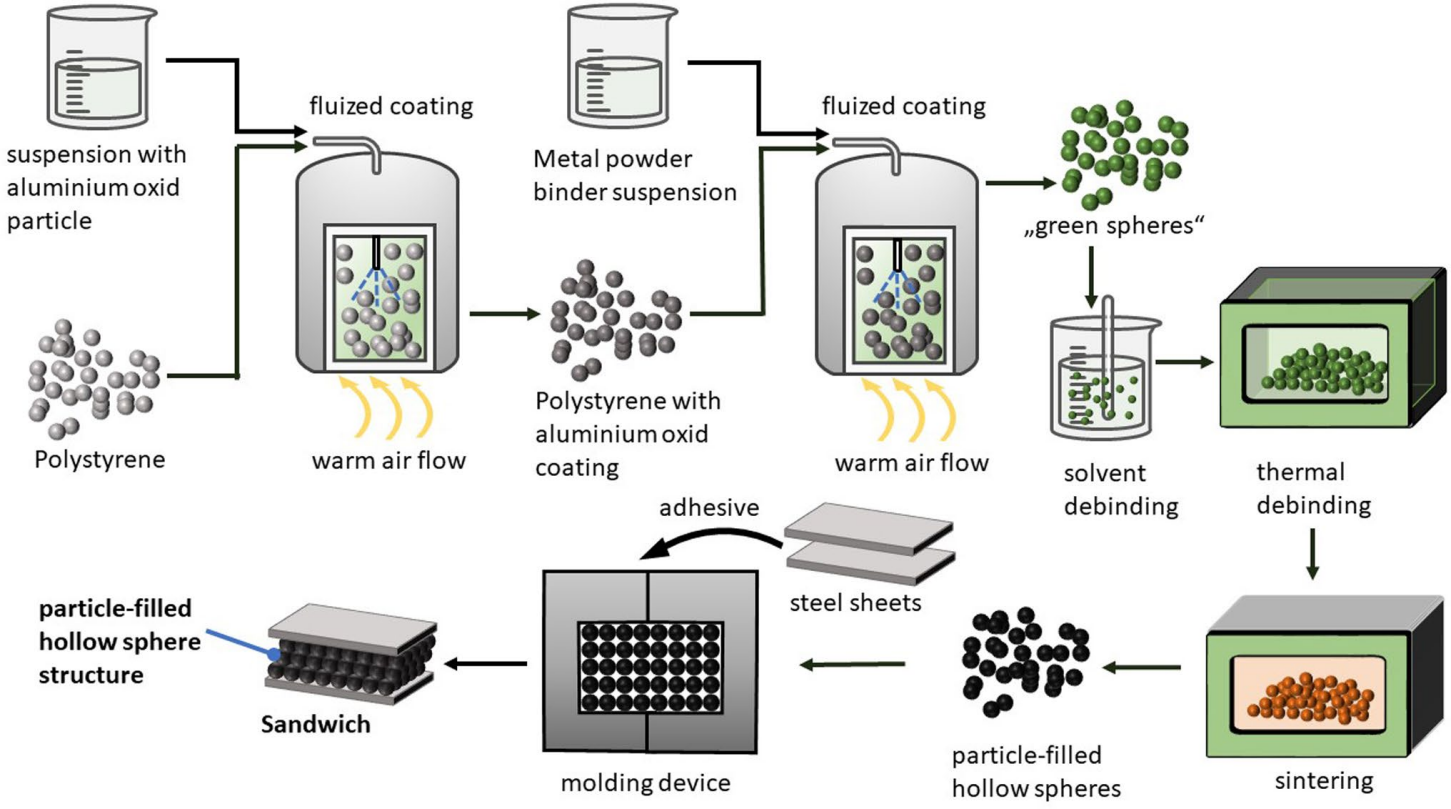
Fabrication process of PHS and PHSS/steel sandwich composite.

The PHSS was fabricated by a method^15^ developed by Fraunhofer IFAM (Fig. 1). Carrier spheres composed of expanded polystyrene (EPS) were subjected to a coating process with purely sintered $\:\alpha\:$ aluminum oxide (Al_2_O_3_ - T60) particles in a fluid bed reactor (the formation of the Al_2_O_3_ particle layer after sintering led to the subsequent filling of particles within the hollow sphere). In this study, high-grade sintered aluminum oxide was selected as the particle filling material for the hollow spheres. The Al_2_O_3_ -T60 has undergone the process of granulation into spherical shapes and has been fused tightly together at temperatures up to 1900 °C. After the sintering process, the material is ground in mills that are lined with Al_2_O_3_. After grinding, the powder consists of large aluminum oxide crystals up to 200 μm in length. As a result, there is a wide range of sizes and irregular forms of particles. The particles are expected to have minimal sintering activity, predominantly closed porosity, exceptional thermal and thermal shock resistance, increased mechanical strength, and commendable chemical resistance. This specific grade also has a significantly low concentration of magnetic iron.

The next step in the manufacturing process is to apply a layer of carbonyl iron powder (the metal powder forms a metallic hollow sphere shell after the next sintering process) and binder to the Al_2_O_3_ layer of these spheres. In addition, a 4% by mass iron phosphide (Fe3P) powder was included to attain a phosphorus concentration of 0.6% by mass in the alloy. Phosphorus enhances the strength of the hollow sphere shell to nearly three times that of pure carbon steel. The increase in strength is achieved by two mechanisms: first, the presence of a temporary liquid phase during sintering greatly reduces the porosity of the shell; and second, the inclusion of phosphorus leads to robust solid solution hardening.

**Fig. 2:**
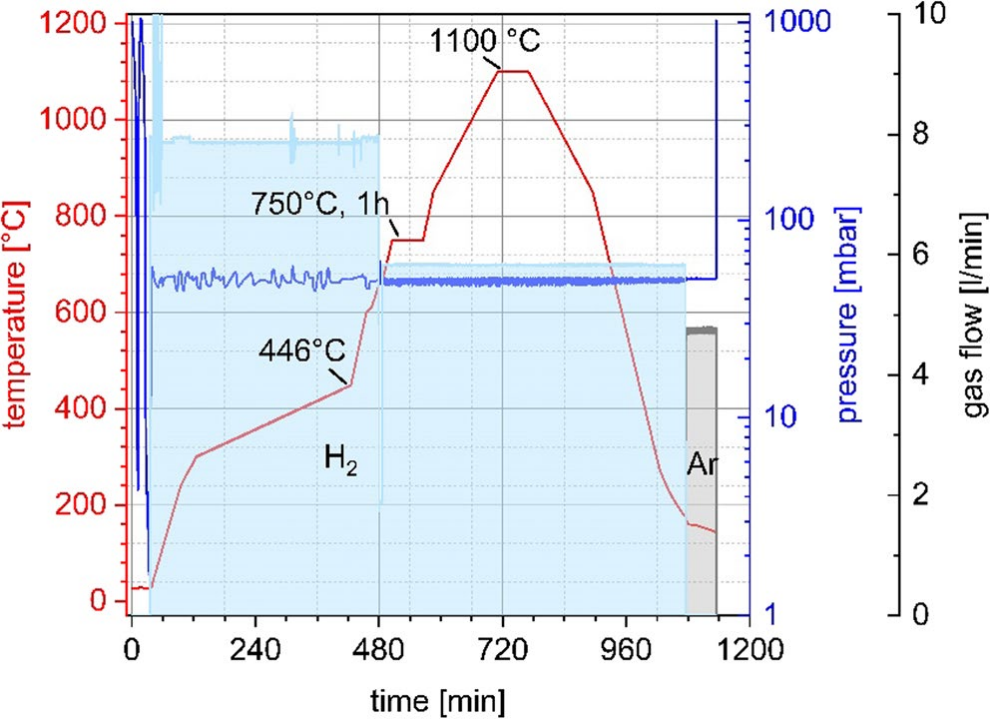
Process parameter for PHS thermal treatment.

The result of this coating process is a pourable substance known as “green spheres”. These green spheres are then debinded with a solvent (acetone) under 23 °C. After an initial solvent debinding process, the next step is a thermal treatment that includes both debinding and sintering. The process parameters are shown in Fig. 2 the including temperature, pressure and gas flow for thermal treatment.

Thermal debinding and sintering of the spheres was carried out in an ISO furnace with rising temperatures and a hydrogen atmosphere at pressure of ca. 50 mbar. For thermal debinding, the temperature was gradually increased to 446 °C within 420 min. Heating for sintering follows debinding without intermediate cooling. Spheres that have undergone thermal debinding but are not yet sintered cannot withstand vibration and pressure fluctuations. The maximum temperature was 1110 °C for the sintering of phosphorus-alloyed steel used in this research. The stage at 750 °C for one hour is necessary to achieve temperature equalization between all points inside the furnace. The advantage of this type of furnace is that it uses a variable gas flow combined with two nested heating zones to eliminate all debinding gases. This process inhibits the formation of carbonaceous deposits on cooler parts of the furnace while removing binders, thus maintaining the cleanliness of the furnace and preventing unintentional absorption of carbon during the sintering process. This design allows debinding and sintering to be conducted in a single furnace, without the need for pressure adjustments or moving the hollow spheres between the two processes. The spheres produced by this process are the PHS.

**Fig. 3:**
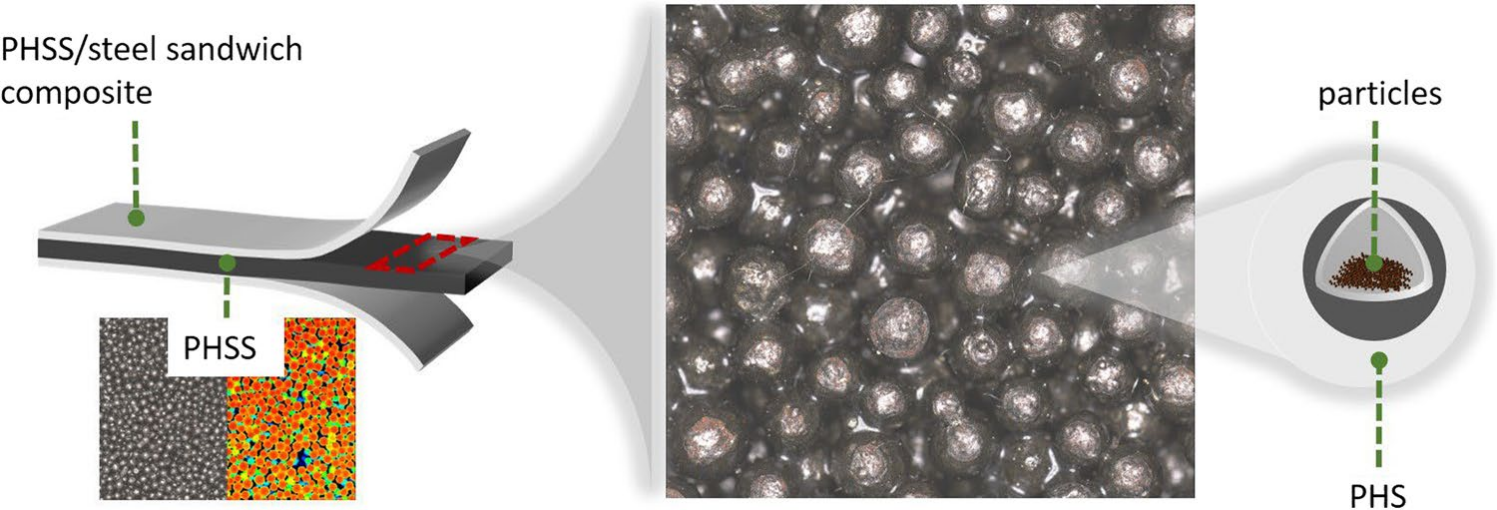
Glue-bonded PHSS in sandwich composite.

Next step is the production of PHSS/steel sandwich composites by bonding two steel cover sheets to the PHSS. The process of bonding was conducted by using one component epoxy adhesive Araldite AT 1–1 (adhesive powder). The PHSs were mixed with binder composition after the thermal treatment without surface treatment and dissolved under low pressure in a specially designed molding device (weighting ca. 1 kg) to form PHSS. The hollow sphere structures were positioned between the corresponding sandwich cover sheets using the molding device. The temperature for the bonding has to be set between 120 and 160 °C to keep a stable networking of PHSs and cover sheets. The complete component (including PHSs, molding device and cover sheets) was then placed in a drying oven with a weight of 650 g on top and chemically bonded at a temperature of 140 °C for 2 h. The adhesive powder melts, wets the surface of the spheres and the sheets and were glued into PHSS/steel sandwich composites demolded after cooling. Glue-bonded hollow spheres can be regarded as a composite material embedded in an air matrix (Fig. 3). The space between the spheres also aids in reducing vibrations.

### Characterization of PHS

In this study, the same batch of EPS was used for all the hollow spheres to ensure that the sphere diameter was approximately the same. Additionally, the thickness of the hollow sphere shell was approximately 40 μm. The average diameter of a PHS is approximately 2 mm. PHS are filled in three different filling ratio (10%, 20%, 25%) and with two particle size distribution, which is classified into T60 0–45 μm and T60 0–200 μm.

**Fig. 4:**
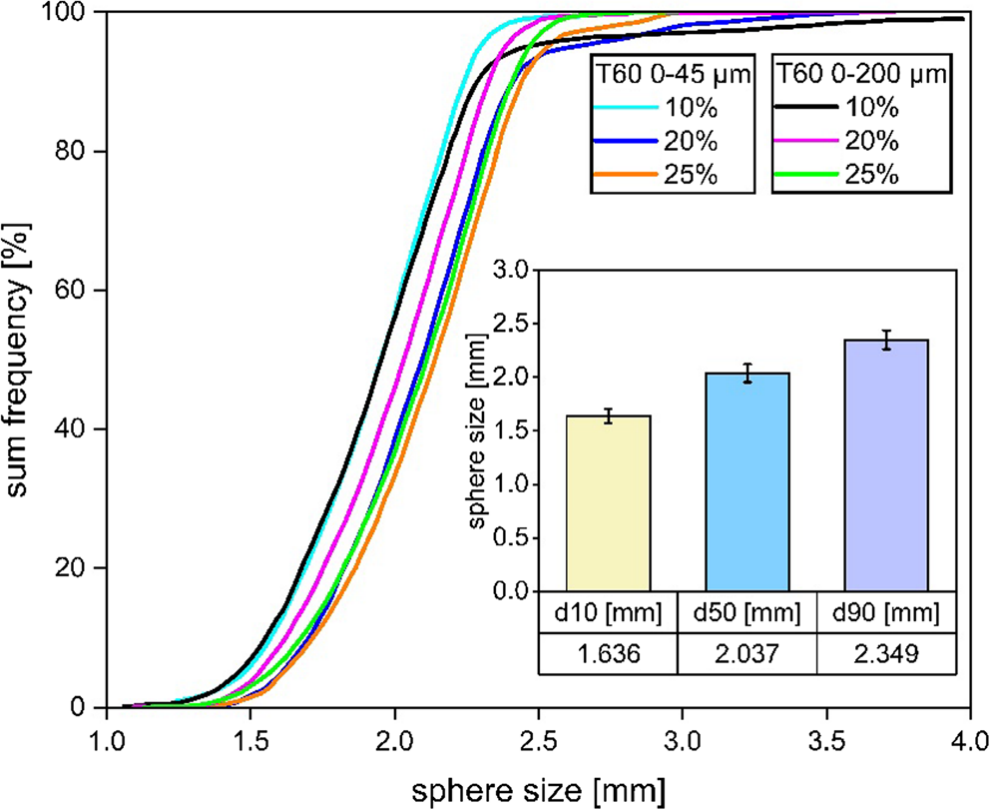
PHS size distribution after sintering.

Figure 4 shows the cumulative PHS size distribution (sum frequency in %) used in this study. A total of six batches of PHS were manufactured, each with a different filling ratio. The PHS size distribution curves for all batches are quite steep, indicating a narrow size distribution around the median sizes. The curves for all batches show similar trend, suggesting that the PHS size distribution is not drastically different with different filling ratios and particle size. The size of the PHS ranges mainly from 1 mm to 2.5 mm, with a d_10_ of 1.636 mm, a d_50_ of 2.037 mm, and a d_90_ of 2.349 mm. The parameters for each batch are shown in Table 1.

**Table 1:** Parameters of manufactured PHS.

Batch	Sphere size [mm]	Wall thickess of PHSS [µm]	Filling ratio [%]	Particle size [mm]
1	2	40	10	T60 0–45 μm
2	2	40	10	T60 0–200 μm
3	2	40	20	T60 0–45 μm
4	2	40	20	T60 0–200 μm
5	2	40	25	T60 0–45 μm
6	2	40	25	T60 0–200 μm

**Fig. 5:**
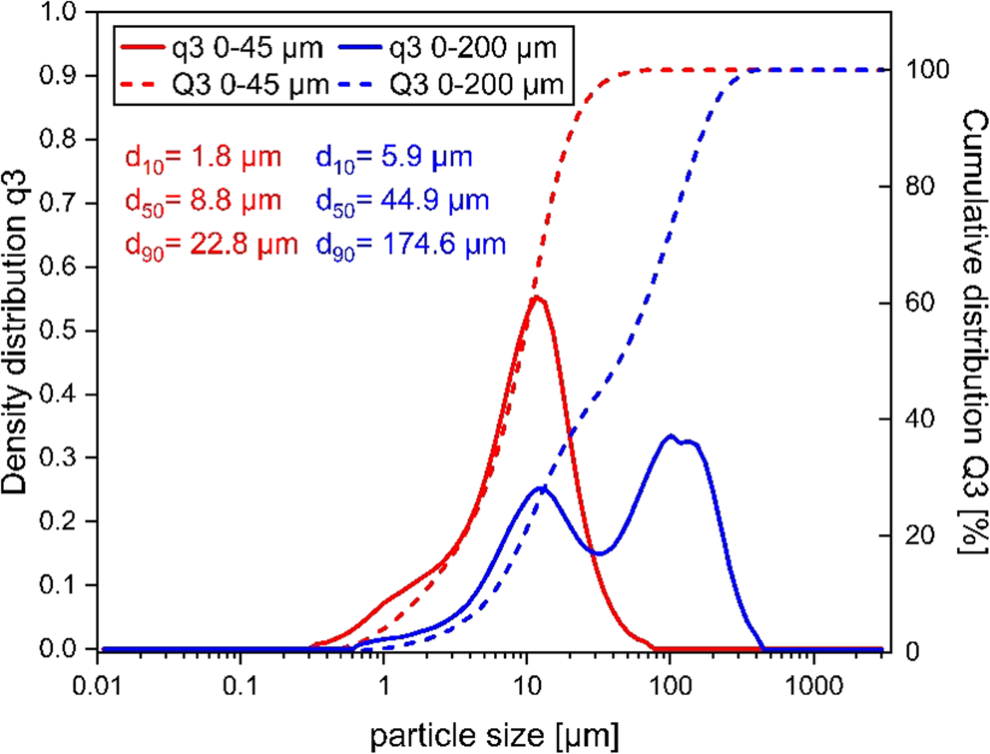
Particle distribution of used aluminum oxide powder T60.

Figure 5 shows the particle size distribution (cumulative distribution Q3 and the probability density function q3) in the ranges 0–45 μm and 0–200 μm for the filling material Al2O3 T60 used in this study. The red curves show the particle sizes distribution from 0 to 45 μm with the Q3 starting at 0 and reaching 100% at about 25 μm, while the q3 peak drops off significantly at 10 μm. The blue curves show the particle sizes distribution from 0 to 200 μm with the Q3 starting at 0 and reaching 100% around 180 μm, while the q3 has multiple peaks: a smaller peak around 10 μm and a larger peak around 100 μm, with additional smaller peaks at higher sizes. It indicates that for the particles in the 0–45 μm range: the particle sizes are more concentrated, with most particles being below 10 μm (d_50_ = 8.8 μm) and this range shows a sharp increase in cumulative distribution, indicating a narrow particle size distribution (d_10_ = 1.8 μm, d_90_ = 22.8 μm). For the 0–200 μm range, the particle sizes are more spread out, showing a broader, more complex, multimodal distribution with d_10_ = 5.9 μm, d_50_ = 44.9 μm and d_90_ = 174.6 μm.

## Characterization of the mechanical properties of PHSS

**Fig. 6:**
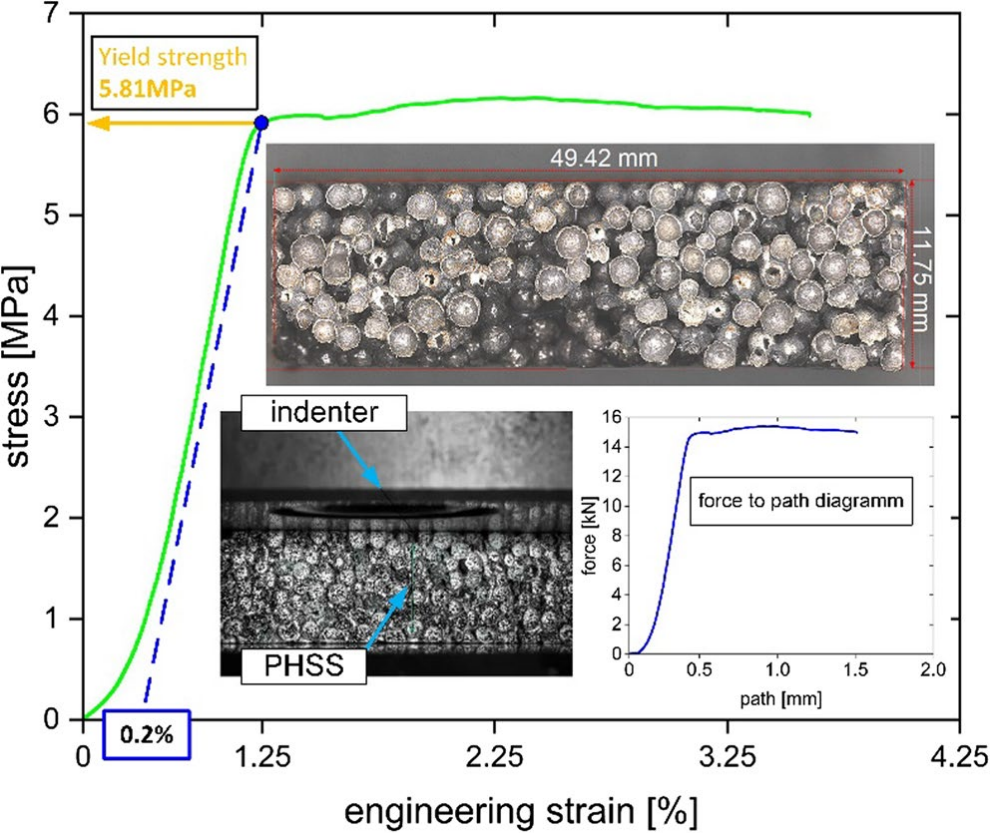
Stress-strain curve from yield strength test.

The compressive yield test was performed to evaluate the mechanical performance of the PHSS. A ca. 10 mm thick specimen with an nominal area of 50 × 50 mm was observed through water jet cutting and used in the experiments. The dimension of this specimen was measured by profilometer setting tangent lines to most PHSs on the surfaces to determine the average length and width (Fig. 6). The stress-strain curve of the PHSS is shown in Fig. 6.

The PHSS has a yield strength of 5.81 MPa. The actual dimensions of the specimens used in the compressive yield test were measured using a profilometer. Due to the cellular structure of PHSS, the boundaries of the material are not perfectly straight, which makes it difficult to measure the exact dimensions of the specimens (Fig. 6). In addition, the surface of PHSS is not planar and the joint between the indenter and the specimen in the compressive test has a non-negligible effect on the estimation of the elastic modulus of the material from the stress-strain curve. Therefore, the accuracy of estimating the elastic modulus of the material directly from the linear elastic portion of the stress-strain curve is limited.

**Fig. 7:**
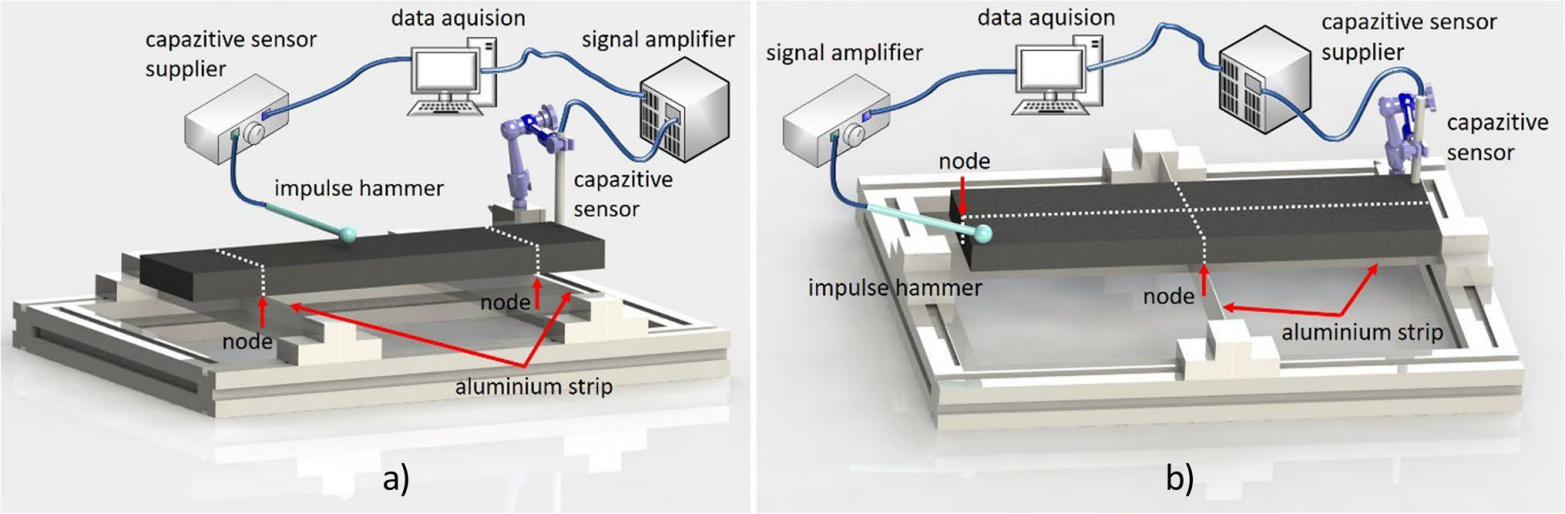
Test rig for IET testing and measured first bending (**a**) and torsional (**b**) frequency.

The Impulse Excitation Technique (IET) is a method used to measure material parameters. It is applicable to most isotropic materials, and it is acceptable for porous materials because it requires only small strains and has few limitations on specimen geometry. Compared to static methods such as tensile testing and flexural modulus testing^23^, IET is a non-destructive testing method that uses a simpler test rig. Nondestructive testing methods using ultrasonic^24^ are not suitable for PHSS because the excited particles and the joint face between PHS disturb the signal to be measured. Figure 7 shows the test fixture developed in this study to perform the IET test on PHSS specimens. Two 1 mm wide aluminum strips were mounted on a platform made of aluminum profiles. The aluminum strips were fixed at the position of the vibration node of the first bending mode of the specimen. It is essential the design the dimension and shape of the tested structure ensuring the first bending and first torsional eigenfrequency are clearly separated to avoid modal coupling in the IET. In the experiment, a test specimen that was 400 mm long, 100 mm wide, 25 mm thick and filled with 10 vol% T60 0–45 $\:\mu\:m$ particles was used and placed on the strips. The material was excited by an impulse hammer with a piezo force transducer (Brüel & Kjær 8200) at the central position, and a capacitive transducer (microSense model 5002) located at the edge of the material was used to measure the response displacement signal. The first bending frequency of the material was obtained through the measured frequency response function (FRF) recorded by the Brüel & Kjær Pulse 3650 signal processing system (Fig. 7a). To measure the first torsional frequency (Fig. 7b), two aluminum strings arranged as a cross were fixed on the platform. They were also positioned at the vibration node of the first torsional mode of the specimen. In this case, the measurement point was located at one end edge of the material, and the excitation point was located at the other end.

**Fig. 8:**
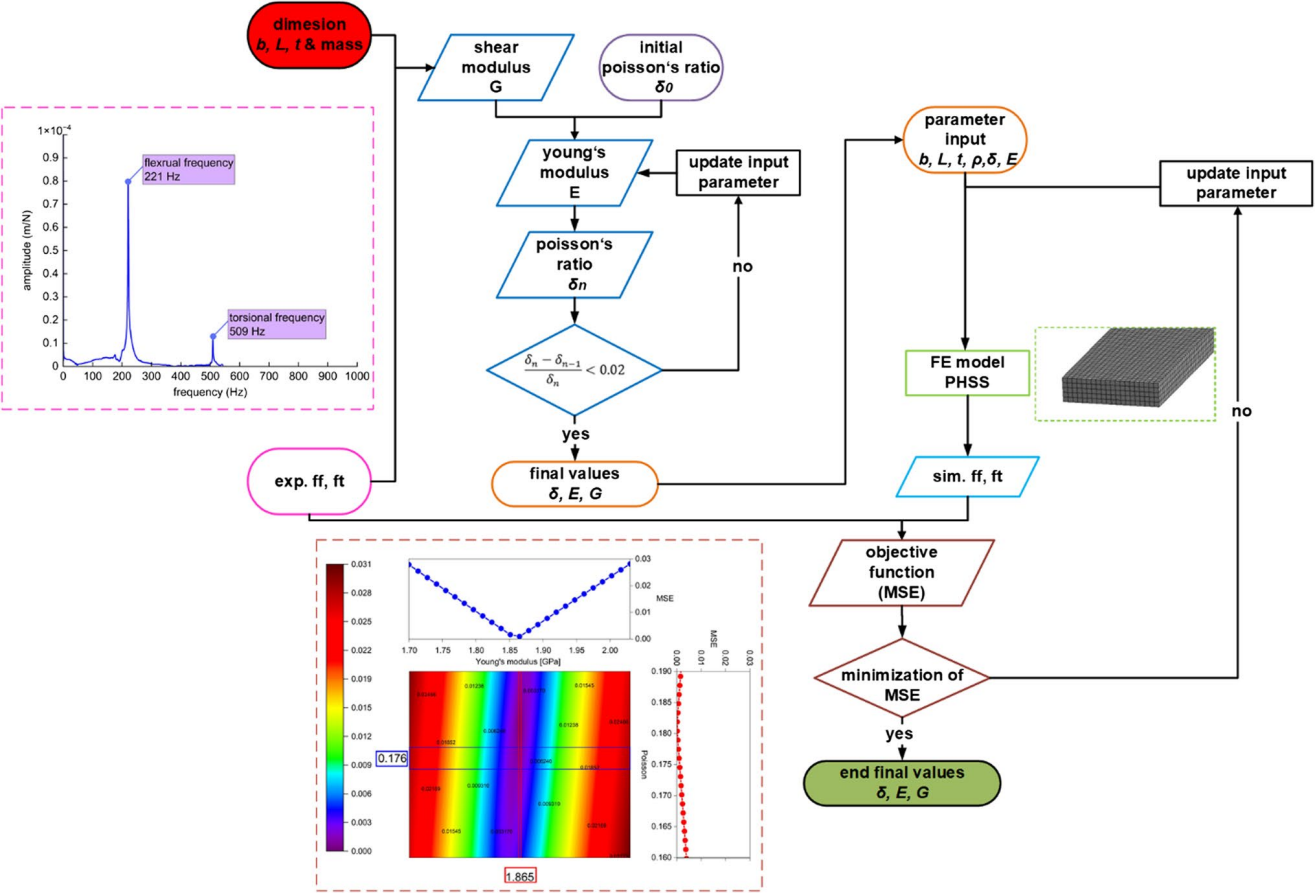
Optimization approach to determine material parameter measured by IET method.

The IET calculation based on measured bending and torsional frequencies is presented in the Appendix. The manufacturing tolerances of PHSS may cause errors in obtaining the exact width and length *b*,* L*, density $\:\rho\:$ by direct measurement, resulting in inaccuracies in the analysis of material properties using the IET approach. To address the potential size and edge effect of PHSS, an optimization approach (Fig. 8) was developed to minimize the effect related to size and improve the accuracy of the IET method in determining material parameters of PHSS.

The optimization approach contains two iterative processes to determine the material parameters of PHSS:

1. Since Young’s modulus E and shear modulus G were both unknown quantities and the ratio of length to thickness of the specimen is 16 smaller than 20, in this study, an iterative model is first required to determine Poisson’s ratio ratio $\:\delta\:$^25^.
2. The standard IET method does not take into account the inaccuracies of the porous material of the specimen, whose manufacturing tolerance is difficult to controlled. The objective of the second iterative process is to minimize the discrepancy between measured and simulated flexural $\:{f}_{f}$ and torsional frequencies $\:{f}_{t}$ in order to obtain the accurate material parameters of PHSS. The simulated results are derived from modal analysis with unconstrained boundaries with a homogenized finite element (FE) model using Ansys. The FE model used solid 186 elements to discretize the layered structure. The identified material parameters using only IET method and additionally using optimization approach are listed in Table 2.

**Table 2:** Material parameters of PHSS determined by standard IET and IET with optimization.

Material parameters	Standard IET	Optimization approach
Young’s modulus [GPa]	1.852	1.865
Shear modulus [GPa]	0.789	0.793
Density [g/cm³]	0.956	0.960
Poisson’s ratio	0.173	0.176

The optimization approach with two iterative processes based on IET method allows efficient determination of multiple unknown material parameters of PHSS by non-destructive testing and takes into account unavoidable manufacturing tolerances. The comparison of IET and optimization approach shows the applicability of IET to PHSS for the material configuration used in this article. The optimization approach presents usability in refining the accuracy of determining PHSS properties further, particular in scenarios where tolerances of material dimensions increase. It offers great potential for the identification of material properties of PHSS with different sphere configurations.

## Design and test of PHSS/steel sandwich composite

The previous work^15^ showed that the size of sphere and the thickness of the sphere shell barely effect the damping characteristics, while the filling ratio and the particle size distribution demonstrated a significant effect on damping. When multiple PHSs are bonded together to create the PHSS and incorporated into a sandwich composite to enhance its stiffness-to-mass ratio, the structural parameters will inevitably influence the damping property of the sandwich composite. Therefore, it is crucial to investigate how the design and particle parameters effect the damping performance of the sandwich composite before they can be effectively utilized in mechanical engineering applications.

**Fig. 9:**
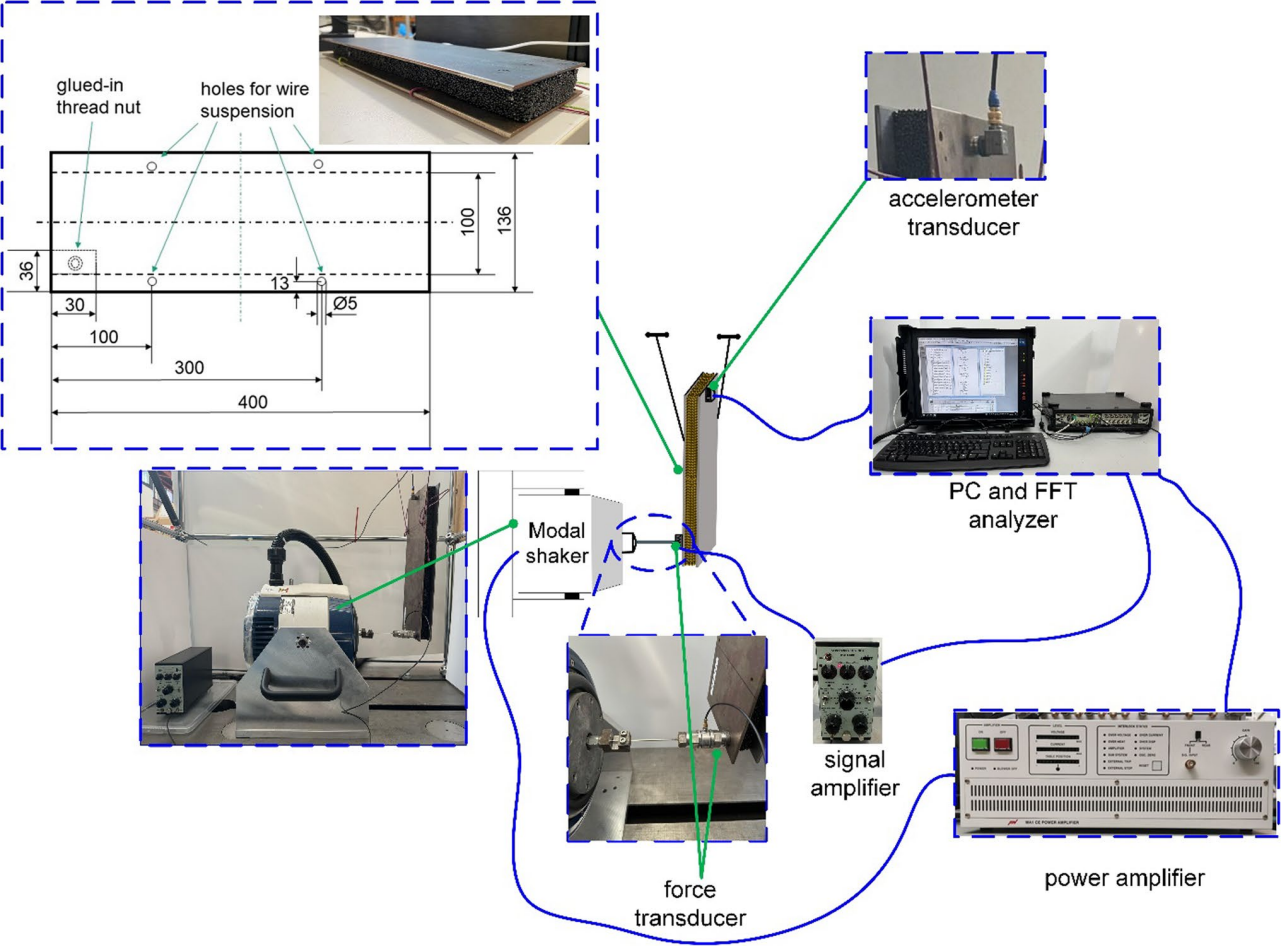
Dimension of investigated PHSS/steel sandwich composite and test rig of its damping characterization.

The PHSS with same length and width as it used for IET test have constructed as core for sandwich composite. In order to simulate the free boundary conditions as closely as possible in the experiments, the cover sheet was designed to be wider than the sandwich structure to provide space for suspending the specimen with ropes. The cover steel sheets made of structural steel have the same length as PHSS and 136 mm width. The suspension holes are located at the vibration nodes of the first bending mode (Fig. 9).

The design of experiment (DoE) aims to determine effects of design parameters (thickness of PHSS and steel cover sheet) and particle parameters (filling ratio of PHS and filled particle size) on the damping performance. The number of experiments as well as the number of material specimens would be very large if full analytical factorization experiments were conducted for all the four parameters under investigation. In order to effectively narrow the scope of the experimental design and ensure that there is a sufficient number of specimens to support the full study of all parameters. Considering the size limitations of the current PHSS in the laboratory production, the number and parameter configuration of test specimens were determined using D-Optimal design approach. The design included varying the four parameters at different levels: thicknesses (10, 15, 25 mm), filling ratios (0%, 10%, 20% and 25%) and two particle size batches (0–45 $\:\mu\:m$ and 0-200 $\:\mu\:m$). To investigate more parameter levels, the test specimens without filling particles (0% filling ratio-unfilled PHS) were also designed. The design matrix of test number is generated in Design-Expert and shown in (Table 3, in Appendix).

### Test rig

In the experiment, a sweep sine signal was generated by a Brüel & Kjær Pulse 3650 signal processing system and boosted by a power amplifier (IMW MA1-CE) to drive the modal shaker (IMW m060-CE) to excite the sandwich specimen. The test specimen was excited by the shaker with a axially rigid stinger system and a Kistler 9321 C force transducer measured the output force applied to the structure. Due to the limitation of non-contact measurements for free-hanging objects, a magnetically mounted accelerometer (Endevco model 66A50) was used to measure the feedback accelerometer signal. HBM was used to determine the damping ratio of the test pieces. To obtain accurate experimental results, the structure was subjected to broadband excitation from 0 to 1600 Hz with a resolution of 0.25 Hz to determine the eigenfrequency. A zoom FFT analysis was then performed near of each eigenfrequency with a bandwidth of 200 Hz and a frequency resolution of 31.25 mHz to accurately calculate the damping ratio. To enhance the reliability of the average result, each specimen was tested five times, allowing for the assessment of consistency and accuracy through statistical analysis.

### Response surface analysis

Response surface analysis was performed on the experimental data and a quadratic mathematical model with interaction terms (Eq. (1)) was applied to investigate the effect of the independent variables (design parameters and particle parameters) and their interaction on the dependent variable (damping ratio).

1 $$\begin{array}{c}{\varvec{Y}}={\beta }_{0}+{\sum_{i=1}^{k}{\beta }_{i}{{\varvec{X}}}_{{\varvec{i}}}}_{1}+\sum_{i=1}^{k}{\beta }_{ii}{{\varvec{X}}}_{{\varvec{i}}}^{2}+\sum_{i=1}^{k}\sum_{j>i}^{k}{\beta }_{ij}{{{\varvec{X}}}_{{\varvec{i}}}{\varvec{X}}}_{{\varvec{j}}}+\upepsilon \\ i=\text{1,2},\dots k;j=\text{1,2},\dots k;i\ne j\end{array}$$

Where $\:\varvec{Y}$ is response (here damping ratio), $\:{\beta\:}_{0}$ is a constant, and $\:{\beta\:}_{i}$,$\:\:{\beta\:}_{ii}$, $\:{\beta\:}_{ij}$ are the linear, quadratic and interaction terms. $\:{\varvec{X}}_{\varvec{i}}$ and $\:{\varvec{X}}_{\varvec{j}}$ are the levels of the independent variables. $\:k$ is the number of independent variables and ${\epsilon}$ is the random-error variable.

**Fig. 10:**
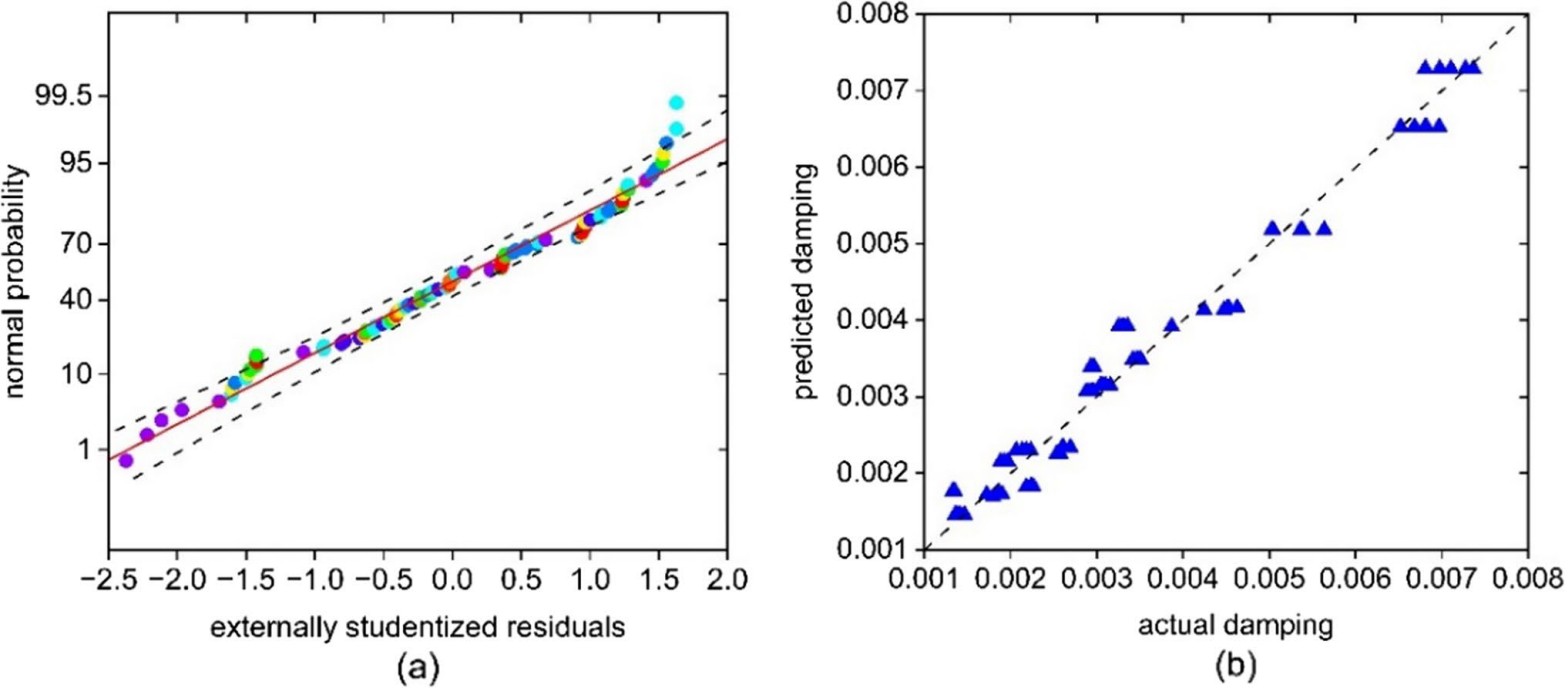
(**a**) Normal probability plot of externally studentized residuals for statistical model and (**b**) model predicted damping with measured damping ratio.

Linear regression is used to test whether the developed mathematical model is a good fit to the actual observations. Figure 10a illustrates an externally weighted residual analysis and a normal probability distribution test used to evaluate the quality of the model fit. It indicates that there are a minimal number of residual outliers, and the general residual distribution is close to a normal distribution. Simultaneously, the predicted damping ratio based on the response surface model shows only a small deviation from the actual measured damping ratio (Fig. 10b), illustrating that the model accurately fits the data.

An F-test shows that the overall fit of the model to the data is statistically significant, with an F-value of 174.3 and a P-value of less than 0.01. This indicates that the model contains many terms that significantly affect the dependent variable.

**Fig. 11:**
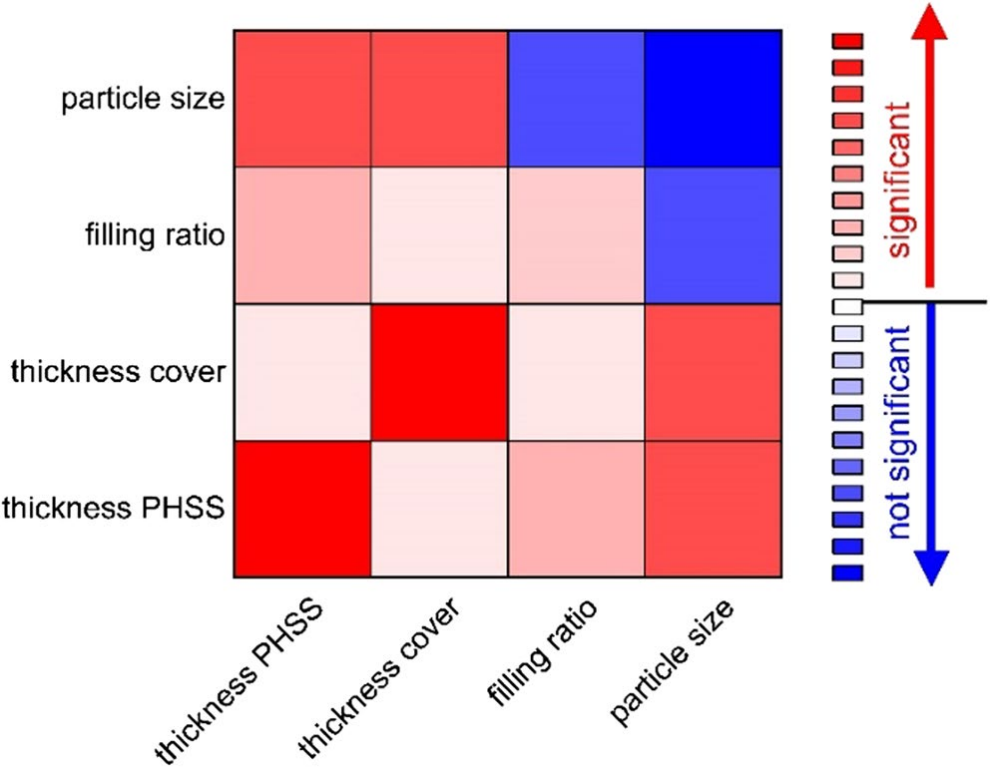
Analysis results for mathematical model.

As shown in Fig. 11, the design parameters (thickness of PHSS core and steel cover sheet) have a significant effect on the damping performance of the sandwich structure. When they are treated as independent variables without considering any interaction with other parameters, the filling ratio has a weak effect, and the particle size distribution does not have a statistically significant impact on the damping performance. The damping is not sensitive to the particle size distribution within the tested ranges. The interactions between the particle size and the design parameters of the PHSS/steel sandwich composite significantly affect its damping performance. It is essential to consider the design parameters as the primary factors and then the particle parameters as the secondary parameters when optimizing the damping of the structure.

**Fig. 12:**
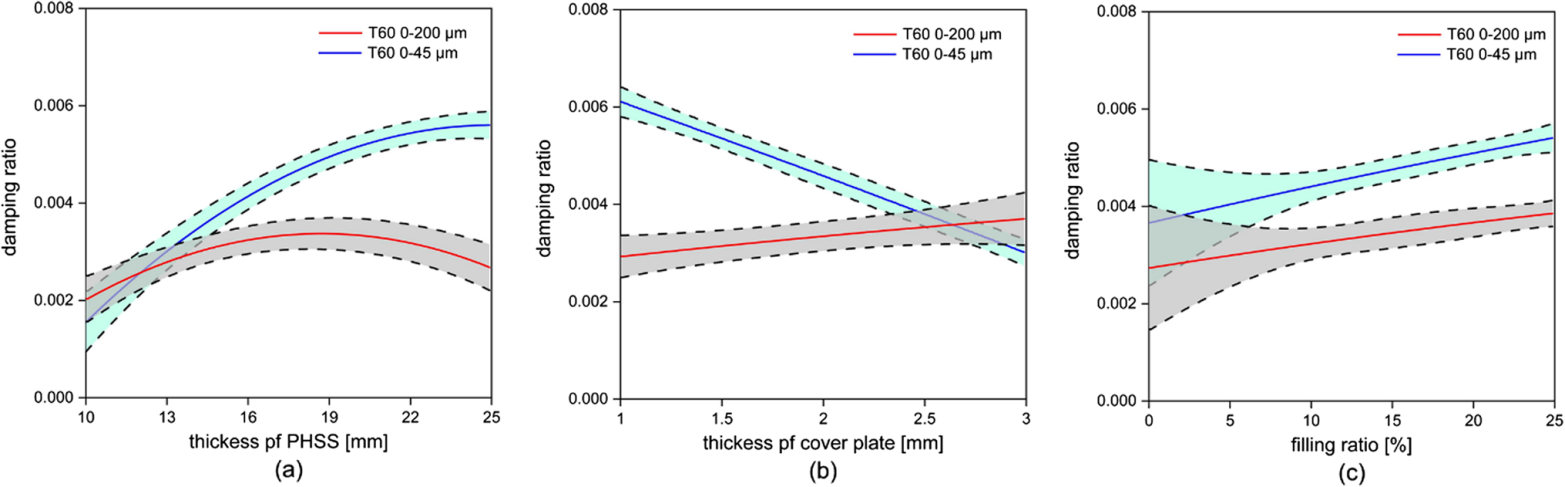
Effects of independent variables on PHSS/steel sandwich composite’s damping performance.

Figure 12 shows quantitatively the effect of the independent variables on the damping performance of the studied PHSS/steel sandwich composite. Figure 12a and b show that for the composite containing particles in the size range of 0–45 μm, the damping performance increases with increasing thickness of the PHSS core, while an increase in the thickness of the cover sheet has an opposite effect. For the composite containing 0–200 μm particle, the damping exhibits an initial increase as the thickness of PHSS increases, followed by a subsequent drop. The thickness of the cover sheet has barely effect on damping. Figure 12c demonstrates that the damping effect rises with an increase in the filling ratio. The discrepancy in the data fitting between the unfilled and filled sandwich composites is attributed to the differences in the manufacturing processes used for each type. The filled composites require the application of two coatings on the base sphere, while the unfilled composites require only one. The factors investigated in this study show significant interactions (Fig. 11), indicating that the influence of multiple parameters on the damping of sandwich composite cannot be analyzed simply by superimposing the effects of each individual parameter.

**Fig. 13:**
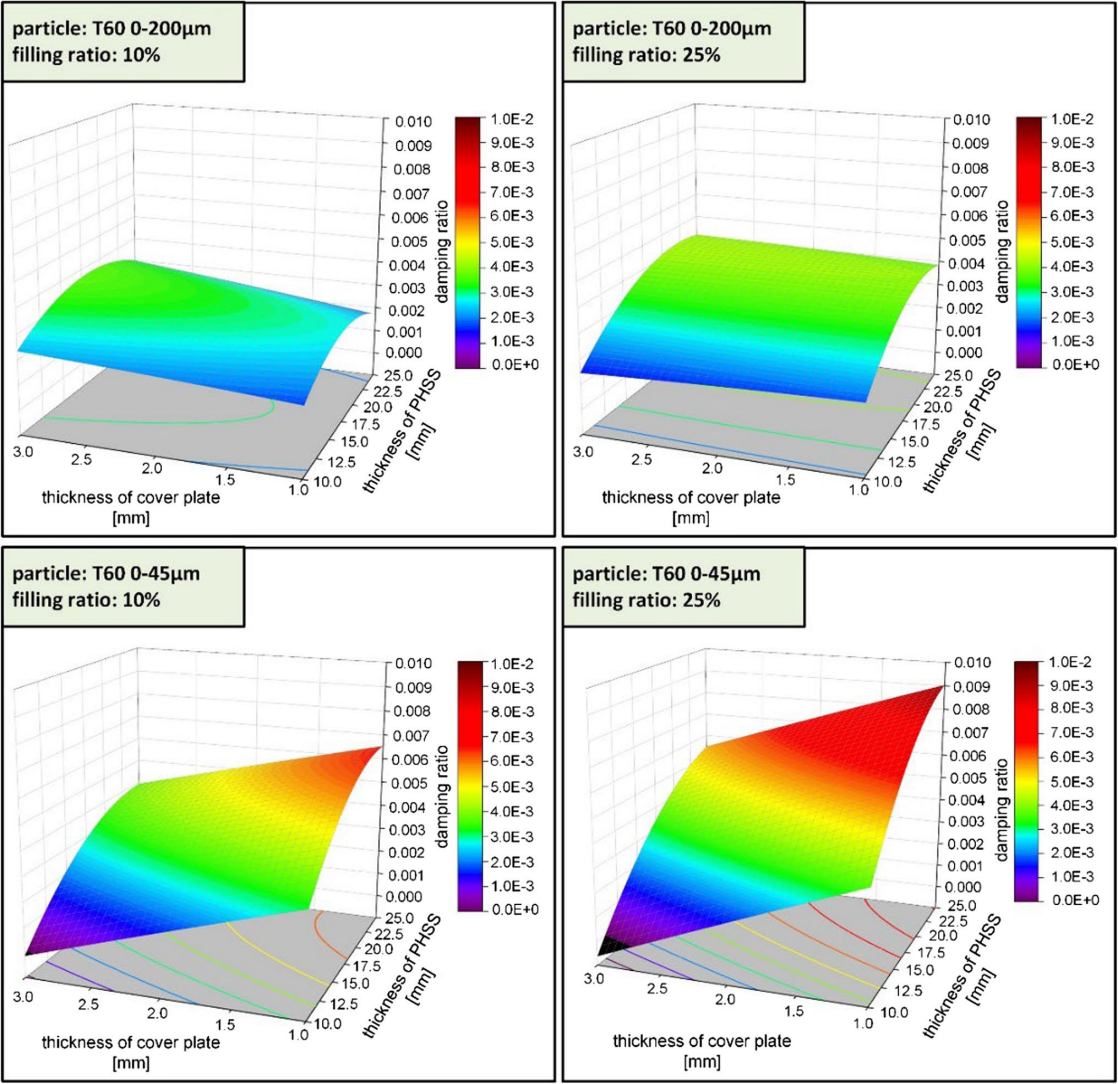
Damping response based on thickness of PHSS and steel cover sheet in different filling ratio and particle size.

Figure 13 shows how the thickness of the PHSS and cover sheets, the filling ratio, and the particle size affect the damping of the sandwich composite. When the PHSS is filled with 10%, 0–200 μm particles, the gradient of the response surface is relatively flat within the range of values examined indicating low damping ratio. The highest damping ratios occur when both the cover sheet thickness and the PHSS thickness are at their maximum values. The increase in damping ratio is modest across the entire range. For the specimens with a filling ratio increased to 25%, the highest values can be observed with thicker cover sheets and PHSS layers, but the overall increase is still relatively modest.

For the sandwich composites filled with 10%, 0–45 μm particles, there is a noticeable increase in the damping ratio, especially for thinner cover sheets and moderate PHSS thickness. The peaks are more pronounced compared to larger particles, indicating better damping performance with smaller particles. For the 25% filling ratio, the 0–45 μm particle size batch shows the highest damping ratios overall. Maximum damping is achieved with 1 mm cover sheets and 25 mm PHSS layers. Increasing the filling ratio and using smaller particles significantly improves damping performance.

It can be summarized that the damping of PHSS/steel sandwich composite is influenced by:

1. Design parameters: Both the thickness of the cover sheet and the PHSS play a critical role on the damping performance of sandwich composite. Thicker PHSS in combination with thinner cover sheets help in optimizing damping performance.
2. Particle parameters: Filling ratio has also significant effect on the damping performance of sandwich composite, but it is relatively weak compared to the design parameters. The particle size distribution within the ranges of 0–45 μm and 0–200 μm does not have a straightforward impact on damping performance of the sandwich composite. The collision effect of large particles tends to be less effective because, for a certain hollow sphere volume, the collision frequency decreases as the number of filling particles decreases which affect the damping characteristics. However, larger particles used in this study have a broader particle sizes distribution, with 50% of the particles being smaller than 45 μm (d50 = 44.9 μm). The smaller particles might fill the gaps between the larger particles during the vibration, resulting in a less pronounced decrease in collision frequency for particles with a large particle size distribution.
3. Interaction between design parameters and particle parameters: The effectiveness of particle size in enhancing damping is highly dependent on the structural properties of the composite, specifically the thickness of the cover sheet and PHSS layer. The combination of design and particle parameters creates a synergistic impact on damping performance: smaller particles (0–45 μm) result in higher damping ratios compared to larger particles (0–200 μm). Increasing the filling ratio from 10 to 25% consistently raises the damping ratio.

## Modeling of PHSS/steel sandwich composite

**Fig. 14:**
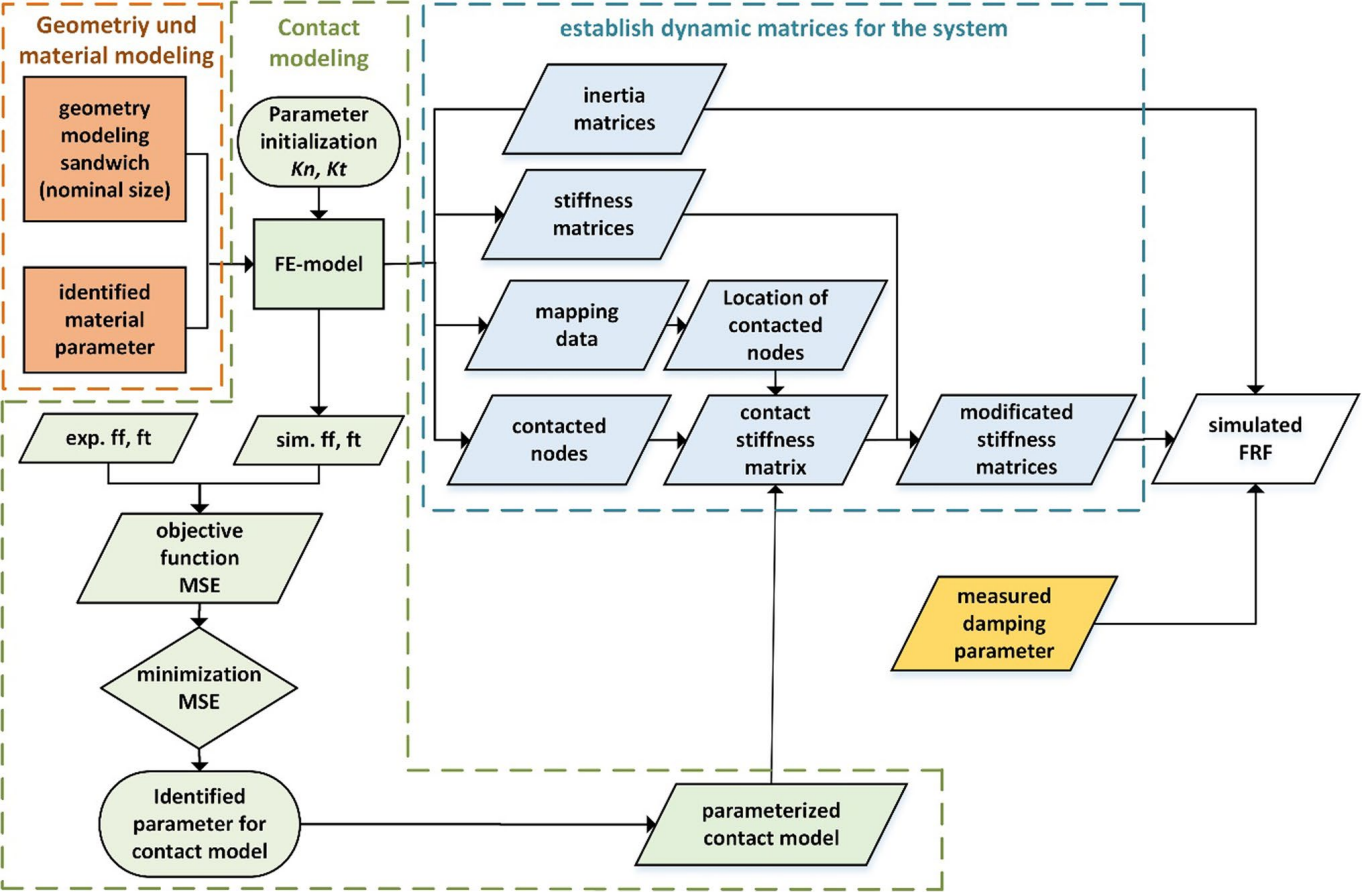
Modeling process of PHSS/steel sandwich composite determining simulated FRF (ff…flexural frequency, ft…torsional frequency, Kn…normal stiffness, Kt…tangential stiffness).

In the design of PHSS/steel sandwich composite and their application in mechanical components, the damping characteristics associated with manufacturing tolerances need to be systematically investigated and reproduced. Modeling strategies based on parameter identification through measurement techniques are important to represent the dynamic properties of PHSS/steel sandwich composite and to eliminate uncertainties. The approach shown in Fig. 14 was used to model the PHSS/steel sandwich composite.

The finite element (FE) model was established in ANSYS consisting of three layers and was discretized with solid elements (solid 186). All layers are modeled as homogeneous materials. The material parameters for steel sheets are taken from literature values^26^, and the parameters for PHSS were determined from the results of the optimization approach shown in Table 2. Since PHSS is a cellular structure, the adhesive layer between PHSS and the steel cover is not considered as a monolithic structure in the fabrication of the composite. Therefore, it is difficult to accurately estimate the thickness of the adhesive layer. Modeling the adhesive layer using the well establish thin-bond-method^27^ leads to significant errors in the estimation of its contact parameters. Hence, an iterative process based on equivalent spring model is used to determine the contact parameters between the sandwich cover sheet and the PHSS.

The initial normal (Kn) and tangential (Kt) contact stiffness of the adhesive layer can be calculated using following functions:

2 $$\:{\varvec{K}}_{\varvec{n}}=\frac{EA}{n{t}_{0}}$$

3 $$\:{\varvec{K}}_{\varvec{t}}=\frac{GA}{n{t}_{0}}$$

where $\:E$ is young’s modulus of used adhesive (Araldite AT 1–1), $\:A$ is the area of adhesive layer subjected to force, *n* is number of equivalent springs for the joint, which was determined by reading the serial number of nodes on the contact surface assigned by ANSYS and saved into mapping data and $\:{t}_{0}$ is initial thickness of adhesive layer.

**Fig. 15:**
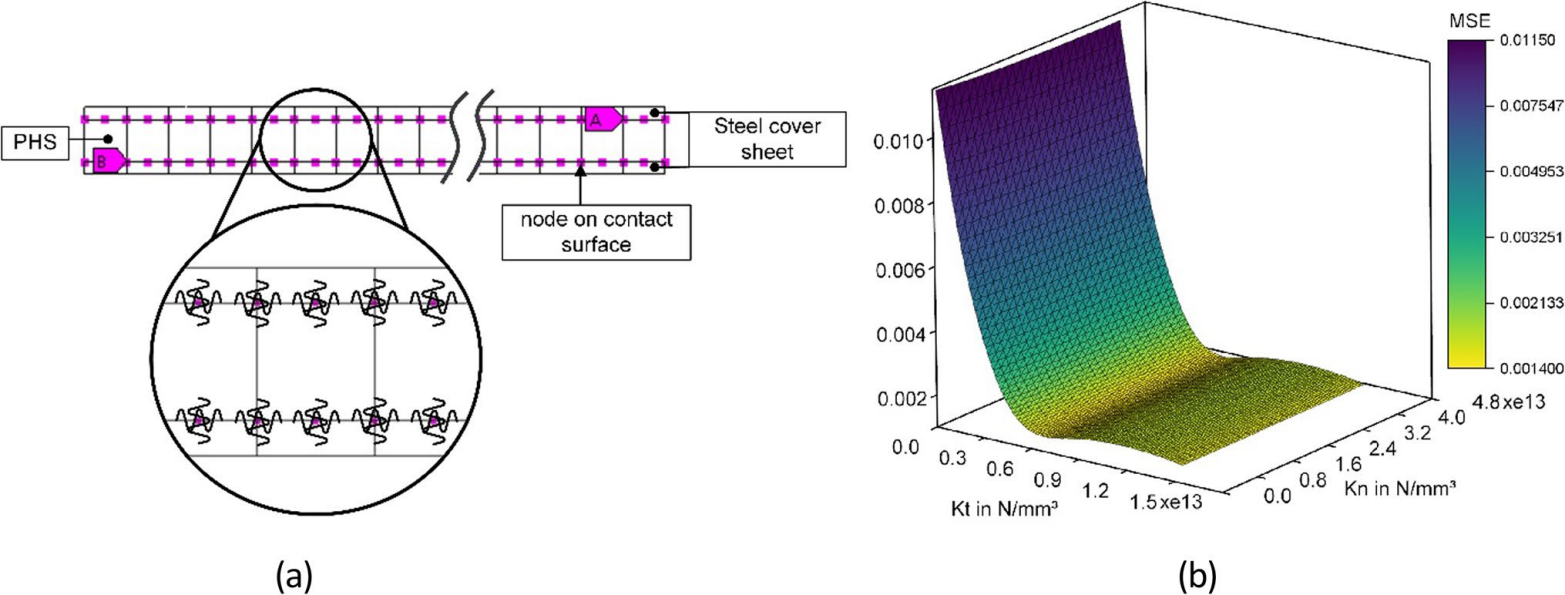
Contact modeling with equivalent spring (**a**) and identified contact paramter for modeling (**b**).

A specimen with a 3 mm cover sheet and a 10 mm core layer filling with T60 0–45 μm particles in 10% was selected to validate the modeling strategy for the sandwich composite. In the iterative process for contact modeling, the FE model is given an initial value of normal and tangential contact stiffness (Fig. 15a) by giving the assuming initial thickness of adhesive layer in 1 mm. These stiffness values were then applied to the FE model to calculate the eigenfrequencies of the sandwich composite. The difference between these frequencies was used to adjust the contact stiffnesses iteratively.

The iterative process minimizes the mean square error (MSD) between the measured bending and torsional frequencies and their simulated values, thereby determining the contact coefficients for the PHSS/steel sandwich composite. Figure 15b shows the identification of contact parameter for the sandwich configuration with 3 mm cover sheet and 10 mm PHSS core.

The stiffness and mass matrix of each layer of the sandwich structure are derived from the established finite element model. Subsequently, the contact parameters observed from the iterative process are used to establish the contact stiffness matrix according to the numbering of the contact nodes on the contact layer. The system stiffness matrix is then constructed based on the individual component matrices and the contact stiffness matrix is added into the system matrix at the position where two nodes are contacted according to the mapping data. Using the measured modal damping ratio of PHSS/steel sandwich composite, along with the acquired mass and stiffness matrix, the measured FRF $\:{\varvec{H}}_{\varvec{m}}$ of the composite is formulated according to the following function.

4 $$\:{\varvec{H}}_{\varvec{m}}=\sum\:_{l}\left(\frac{{\varphi\:}_{R,l}\cdot\:{\varphi\:}_{E,l}}{{{\omega\:}_{l}}^{2}-{{\Omega\:}}^{2}+2i\cdot\:{\vartheta\:}_{l}\cdot\:{\omega\:}_{l}\cdot\:{\Omega\:}}\right)$$

where $\:{\varphi\:}_{R,l}$ is deformation of response point of vibration mode $\:l$ and $\:{\varphi\:}_{E,l}$ ist response of excitation point. $\:{\omega\:}_{l}$ is eigenfrequency of vibration mode $\:l$ and $\:{\Omega\:}$ is excitation frequency. $\:i$ is complex value, $\:{\vartheta\:}_{l}$ is modal damping ratio of the sandwich structure for vibration mode $\:l$.

To ensure the acquisition of an accurate FRF for the sandwich composite, it is necessary to eliminate the influence of the extra mass caused from test configuration (placement of the force sensor with the stinger and the accessible transducer) on the dynamic properties of the test specimens.

The dynamic stiffness matrix $\:{\varvec{K}}_{\varvec{m}}$ with transducers of the measured system can be written into:

5 $$\:{\varvec{K}}_{\varvec{m}}=\varvec{K}-{\omega\:}^{2}(\varvec{M}+{\varvec{M}}_{\varvec{t}})$$

Where $\:\varvec{K}$ is stiffness matrix after correction, $\:\varvec{M}$ is mass matrix of sandwich structure and $\:{\varvec{M}}_{\varvec{t}}$ is mass of transducers.

The FRF of measured system with transducers $\:{\varvec{H}}_{\varvec{m}}$ and the corrected FRF $\:\varvec{H}$ can be expressed as:

6 $$\:{\varvec{H}}_{\varvec{m}}=\frac{{\varvec{X}}_{\varvec{m}}\left(\omega\:\right)}{\varvec{F}\left(\omega\:\right)}=\frac{1}{{\varvec{K}}_{\varvec{m}}}$$

7 $$\:\varvec{H}=\frac{\varvec{X}\left(\omega\:\right)}{\varvec{F}\left(\omega\:\right)}=\frac{1}{\varvec{K}-{\omega\:}^{2}\varvec{M}}$$

Where $\:{\varvec{X}}_{\varvec{m}}(\omega\:$) is measured displacement $\:\varvec{X}(\omega\:$) is corrected displacement and $\:\varvec{F}\left(\omega\:\right)$ is the excitation force in complex value.

So that the relationship between measured and corrected FRF $\:\varvec{H}\left(\omega\:\right)$ is:

8 $$\:\frac{1}{\varvec{H}}=\frac{1}{{\varvec{H}}_{\varvec{m}}}+{\omega\:}^{2}{\varvec{M}}_{\varvec{t}}$$

And $\:\varvec{H}\left(\omega\:\right)$ is simplified as:

9 $$\:\varvec{H}=\frac{{\varvec{H}}_{\varvec{m}}}{1+{\omega\:}^{2}{\varvec{M}}_{\varvec{t}}{\varvec{H}}_{\varvec{m}}}$$

**Fig. 16:**
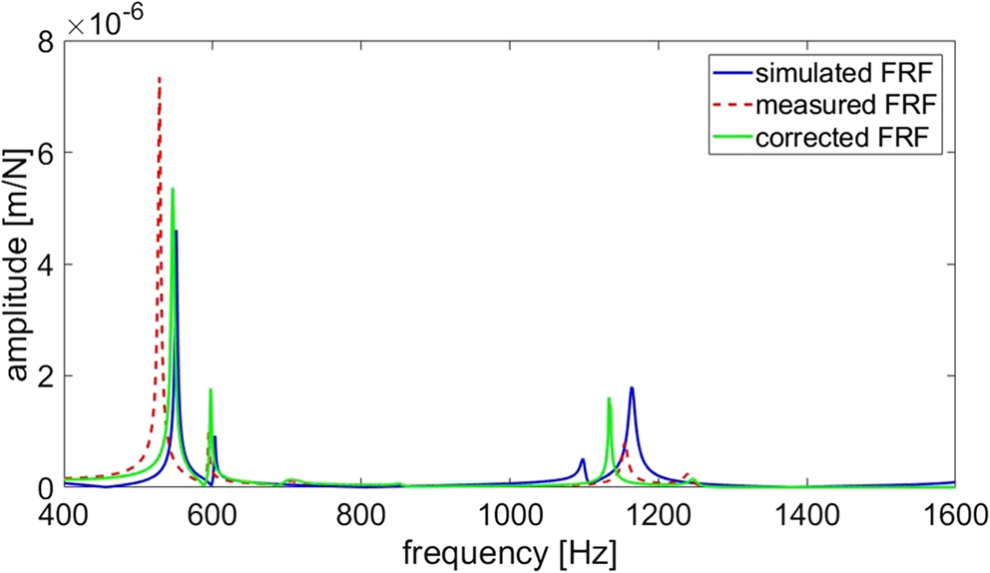
Comparison of simulated, measured and corrected FRF of PHSS/steel sandwich composite.

As shown in Fig. 16, for the first bending and torsional modes, the simulated results closely match the actual corrected FRF. Starting from the third order of natural frequency, the accuracy of the model decreases, which indicates that the filled particles in PHSS exhibit more complex energy transfer and dissipation mechanisms in higher order modes. The increase of structural nonlinearity causes a decrease in accuracy of the linear damping model in simulating its dynamic characteristics. To improve the model accuracy, more refined numerical methods and model parameter identification approaches are required.

## Conclusion

This paper presents a parameter identification method based on the measurement data from IET and simulation using homogeny material model, showing great potential in determining the accurate material parameter of PHSS considering unavoidable manufacturing uncertainties.

The thickness of PHSS and the steel cover sheet (design parameters), and the interaction between these design parameters and particle parameters, significantly affect the damping performance of PHSS/steel sandwich composites. The construction parameters of the PHSS/steel sandwich composite have a more significant influence on damping performance compared to particle parameters. For the design of the PHSS/steel sandwich, it is crucial to consider the design parameters as key factors and optimize the particle parameters to improve the damping performance of the composite. In the sandwich composite, the steel cover sheets provide the primary stiffness while the PHSS experiences primarily the shear deformation. Although the freely movable particles within the spheres contribute to energy dissipation, their effectiveness is still influenced by how much the PHSS deforms under vibration load, which is determined by the sandwich composite construction parameters. A thicker core and thinner cover sheets allow for more deformation, potentially increasing the energy dissipation and optimizing the damping performance of the sandwich composites, which can be further enhanced by decreasing the particle size contribution.

The use of an unconstrained test rig and the application of a correction model to modify the frequency response function acquired from the measurements effectively minimizes the impact of the boundary and test configuration on the measured dynamic properties.

The modeling strategy developed in this study along with linear damping model performs well in investigating the dynamic properties of PHSS/steel sandwich composite in low vibration modes. Due to more complex damping mechanisms and increased nonlinear behavior of the material in higher vibration modes, the model accuracy is limited. Future work should aim at optimizing the damping model for the analysis of PHSS/steel sandwich composite.

The difference in the uniformity of particle size distributions and the overlap in the effective particle size ranges might mask the effects of particle size as an individual factor in the response surface analysis. The broad distribution in the 0–200 μm range (including 50% particle smaller than 45 μm) can introduce variability that makes it difficult to isolate the effect of particle size on damping performance. The further investigation of this phenomenon should potentially isolate the effect of particle size: test narrower particle size ranges for larger particles e.g. 50–200 μm to accurately analyze the effect of particle size for the damping.

This investigation has focused on standardizable test specimens, allowing us to systematically study and identify parameters under certain conditions. The future work aims to transfer and validate these models in real machine part for reliability of the research outcomes in practical application.

## Data Availability

All data generated or analysed during this study are included in this published article.

## Appendix

According to^25^, the young’s modulus $\:E$ of the material specimen can be expressed as,

$$\:E=0.9456\cdot\:\frac{m\cdot\:{{f}_{f}}^{2}}{b}\cdot\:\frac{{L}^{3}}{{t}^{3}}\cdot\:{T}_{1}$$

where *m* is mass, *b* is width, *L* is length and *t* is thickness of the specimen. $\:{f}_{f}$ is fundamental flexural resonance frequency of the specimen. $\:{T}_{1}$ is correction factor for flexural mode to account for thickness and Poisson’s ratio $\:\delta\:\:$. $\:{T}_{1}$ can be calculated with the function below:

$$\begin{array}{c}T_1=1+6.585\cdot\left(1+0.0752\mu+{0.8109\delta}^2\right)\cdot\left(\frac tL\right)^2-0.868\cdot\left(\frac tL\right)^4\\-\left[\frac{8.340\left(1+0.2023\delta+2.173\delta^2\right)\cdot\left(\frac tL\right)^4}{1.000+6.338\left(1+0.1408\delta+1.536\delta^2\right)\cdot\left(\frac tL\right)^2}\right]\end{array}$$

The shear modulus $\:G$ can be expressed as,

$$G=\frac{4Lmf_t^2}{bt}\cdot\left[\frac B{1+A}\right]$$

Where A and B are correction factor dependent on the width to thickness ratio of the test specimen.

$$\:A=\left[\frac{0.5062-0.8776\left(\frac{b}{t}\right)+0.3504{\left(\frac{b}{t}\right)}^{2}-0.0078{\left(\frac{b}{t}\right)}^{3}}{12.03\left(\frac{b}{t}\right)+9.892{\left(\frac{b}{t}\right)}^{2}}\right]$$

$$\:B=\left[\frac{\frac{b}{t}+\frac{t}{b}}{4\left(\frac{t}{b}\right)-2.52{\left(\frac{t}{b}\right)}^{2}+0.21{\left(\frac{t}{b}\right)}^{6}}\right]$$

Poisson’s ratio $\:\delta\:\:$ can be calculated with the relationship in following function

$$\:\delta\:\:=\frac{E}{2G}-1$$

**Table 3:** Sandwich specimens used in the tests.

Thickness PHSS [mm]	Thickness steel [mm]	Filling ratio [%]	Particle size [um]
25	3	25	0-200
10	1	25	0-200
15	2	10	0-200
10	3	10	0-200
10	3	25	0-200
25	1	20	0-200
15	1	25	0-200
10	3	20	0-200
15	3	20	0–45
10	1	20	0–45
15	3	25	0–45
25	3	10	0–45
25	1	10	0–45
15	1	10	0–45
25	2	25	0–45
15	2	20	0–45
10	1	0	
15	2	0	
25	3	0	
